# Generation and Characterization of Drug-Resistant Influenza B Viruses Selected In Vitro with Baloxavir Acid

**DOI:** 10.3390/pathogens11091048

**Published:** 2022-09-15

**Authors:** Amel Saim-Mamoun, Yacine Abed, Julie Carbonneau, Guy Boivin

**Affiliations:** Research Center in Infectious Diseases of the CHU de Québec-CHUL and Laval University, Québec City, QC G1V 4G2, Canada

**Keywords:** Influenza B, baloxavir, antiviral resistance, PA, I38T substitution

## Abstract

Baloxavir marboxil (BXM) is an antiviral drug that targets the endonuclease of the influenza polymerase acidic (PA) protein. Antiviral resistance, mainly mediated by the I38T PA substitution, readily occurs in both A(H1N1) and A(H3N2) viruses following a single dose of BXM. Influenza B resistance to BXM remains poorly documented. We aimed to generate baloxavir-resistant contemporary influenza B/Yamagata/16/1988- and B/Victoria/2/1987-like viruses by in vitro passages under baloxavir acid (BXA) pressure to identify resistance mutations and to characterize the fitness of drug-resistant variants. Influenza B/Phuket/3073/2013 recombinant virus (rg-PKT13, a B/Yamagata/16/1988-like virus) and B/Quebec/MCV-11/2019 (MCV19, a B/Victoria/2/1987-like isolate) were passaged in ST6GalI-MDCK cells in the presence of increasing concentrations of BXA. At defined passages, viral RNA was extracted for sequencing the PA gene. The I38T PA substitution was selected in MCV19 after six passages in presence of BXA whereas no PA change was detected in rg-PKT13. The I38T substitution increased the BXA IC_50_ value by 13.7-fold in the MCV19 background and resulted in reduced viral titers compared to the wild type (WT) at early time points in ST6GalI-MDCK and at all time-points in human epithelial cells. By contrast, the I38T substitution had no impact on MCV19 polymerase activity, and this mutation was genetically stable over four passages. In conclusion, our results show a similar pathway of resistance to BXA in influenza B viruses highlighting the major role of the I38T PA substitution and suggest that I38T may differently impact the fitness of influenza variants depending on the viral type, subtype, or lineage.

## 1. Introduction

Seasonal influenza is a highly contagious respiratory disease with significant public health and economic impacts. Each year, approximately 1 billion cases of influenza infections are recorded in the world [1]. Although most people infected with seasonal influenza strains develop a benign illness, young children, elderly people, and people with chronic medical conditions are at a higher risk of developing serious complications, including death. A typical influenza season results in 3–5 million severe cases and between 290,000 and 650,000 deaths worldwide [2]. Such annual epidemics currently involve influenza A(H3N2) and A(H1N1) strains in addition to influenza B viruses (IBV) which comprise two genetic lineages (B/Victoria/2/1987-like and B/Yamagata/16/1988-like viruses). Different strains from the above subtypes or lineages can co-circulate each year among the population and are associated with various clinical impacts. IBVs are generally less prevalent than influenza A viruses (IAV) in seasonal epidemics, accounting for 23% of total annual cases [3]. However, in some seasons, such as in 2017–2018, IBV infections can represent more than 50% of total influenza cases [4]. While the rates of hospitalization and mortality are similar between influenza A and B infections in the overall population, influenza B viruses may cause more severe disease than influenza A viruses in children. A Canadian retrospective study involving 12 pediatric hospitals from 2004 to 2013 showed that mortality was greater in hospitalized children with influenza B infections than that of children with influenza A infections [5].

Antiviral drugs can provide important clinical benefits for severe cases of influenza A and B infections. The neuraminidase (NA) inhibitors have constituted the main class of anti-influenza agents over the last two decades, including oral oseltamivir (the most widely prescribed NAI compound), zanamivir, peramivir, and laninamivir [6]. NAIs have shown efficacy in the prevention and treatment of influenza infections; however, there is a need to institute NAI therapy within 48 h after the onset of symptoms to obtain significant clinical benefits. Of importance, Japanese clinical studies have shown that oseltamivir is less effective at treating influenza B than influenza A infections [7,8].

Baloxavir marboxil (BXM) is a novel RNA polymerase inhibitor that has been approved for the treatment of uncomplicated influenza in the USA and Japan in 2018 and in Europe in 2021. Its active compound, baloxavir acid (BXA), targets the cap-dependent endonuclease activity located within the influenza A and B polymerase acidic (PA) proteins, thus inhibiting viral mRNA synthesis and therefore blocking viral replication [9]. BXM affords attractive properties for clinical use, including single-dose oral administration. In clinical trials, BXM conferred a greater reduction in the median duration of viral shedding than oseltamivir (24 h vs. 72 h) [10]. However, BXM demonstrated a low genetic barrier to the development of drug resistance. Indeed, BXM-resistant variants were detected in 18 out of 77 (23.3%) children who received BXM therapy [11]. The reduced susceptibility phenotype of these variants mainly involves amino acid substitutions at residue 38 (I38T/M/F) of the PA protein as well as other PA changes, such as E23K and A37T [11]. Among these substitutions, the I38T is a major marker of resistance in influenza A(H3N2) and A(H1N1) viruses. Notably, the I38T change has been detected in an influenza A virus recovered from a hospitalized Japanese child without BXM therapy [12], which suggests the potential dissemination of influenza viruses with reduced susceptibility to baloxavir [13,14]. Our group and others previously investigated the impact of the I38T substitution on the viral fitness of various influenza A(H1N1) and A(H3N2) strains [11,15,16]. Nevertheless, the characterization of baloxavir resistance in IBV remains less extensively investigated than in IAV.

The major objective of this study was to induce antiviral resistance in IBV for the two influenza B/Victoria/2/1987-like and B/Yamagata/16/1988-like lineages by passaging the viruses under BXA pressure in order to identify which mutation could occur under BXA pressure in influenza B viruses and to predict whether such mutation may alter the in vitro properties of eventual variants.

## 2. Results

### 2.1. Selection of Influenza B/Victoria/2/1987-like I38T PA Variant after In Vitro Passages with BXA

Viruses from the B/Victoria/2/1987 lineage (MCV19 isolate) and B/Yamagata/16/1988 lineage (rg-PKT13) were serially passaged under BXA pressure with increasing drug concentrations. Mutations in the PA gene were investigated at different passages in presence or absence of the drug. The B/Victoria/2/987-like MCV19 isolate showed evident cytopathic effects in presence of increasing concentrations of BXA. The PA-I38T substitution was detected in MCV19 after six serial passages in ST6GalI-MDCK cells corresponding to 200 nM of BXA. No additional substitutions were found in other viral genes (NP, PB1, PB2, NA, HA, M, and NA genes). There were no mutations in the PA gene of the control viral sample recovered after six passages without BXA. Contrasting with MCV19, the B/Yamagata/16/1988-like rg-PKT13 virus showed weaker cytopathic effects and some passages needed to be repeated at the same BXA concentration. We lost the PKT13 virus in presence of 100 nM and could not identify new mutations (by Sanger sequencing) at lower BXA concentrations. 

### 2.2. Impact of I38T Substitution on the Phenotypes of Susceptibility to BXA in IBV Isolates and Recombinant Viruses

The mean BXA IC_50_ values for the B/Victoria/2/1987-like MCV19 and rg-B/Washington/02/2019 (a B/Victoria/2/1987-like recombinant virus rescued with reverse genetics using the contemporary vaccine strain) viruses and their PA-I38T variants are reported in Table 1. The PA-I38T substitution increased the IC_50_ values by 13.7-fold and 21.3-fold in MCV19 and rg-B/Washington/02/2019 backgrounds, respectively. For comparison, the PA-I38T substitution was shown to increase the IC_50_ value of the rg-PKT13 virus by 12.6-fold [13].

### 2.3. Impact of the PA-I38T Substitution on In Vitro Replication Capacities of MCV19 Isolate in ST6GalI-MDCK Cells

**Figure 1:**
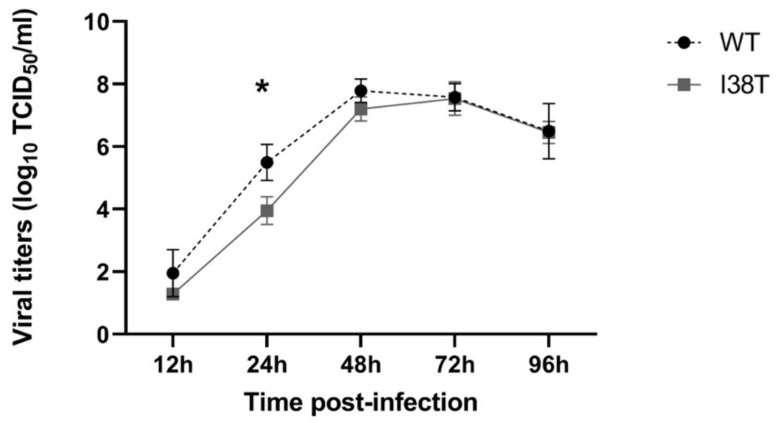
Replication kinetics of MCV19 WT and its I38T variant in ST6GalI-MDCK cells. Confluent ST6GalI-MDCK cells were infected with an MOI of 0.0001. Supernatants were collected at the given time points and titrated by TCID_50_. Mean viral titers of triplicates ± standard error of the mean were determined as TCID_50_/mL. The mean of three independent experiments was calculated. * *p* < 0.05.

In replicative capacity experiments performed on ST6GalI-MDCK cells, the viral titers for both MCV19 WT and I38T viruses reached a peak at 48 h p.i. with mean viral titers of 7.8 and 7.2 log TCID_50_/mL, respectively (Figure 1). A significant difference in viral growth titers between the two viruses was only observed at 24 h p.i. where MCV19 WT and its PA-I38T variant grew at mean titers of 5.49 and 3.95 log TCID_50_/mL, respectively (*p* < 0.05).

### 2.4. Impact of PA-I38T Substitution on In Vitro Replication Capacities of MCV19 and rg-PKT13 Viruses in Human Nasal Airway Epithelium

**Figure 2:**
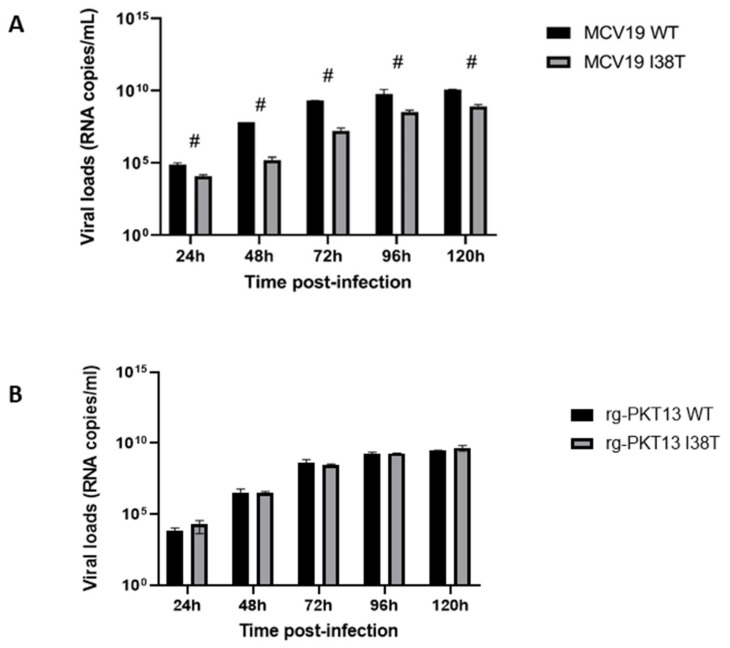
Replication kinetics of MCV19 and rg-PKT13 WT and I38T variants on human nasal airway epithelium. Infections were performed at the apical pole of HAE at an MOI of 0.002 for each virus. (**A**) MCV19 WT and its I38T variant generated by passaging (**B**) rg-PKT13 and its I38T variant generated by reverse genetics. Two experimental conditions were performed and viral loads were determined by qRT-PCR in triplicates for each of the two conditions and analyzed with *t*-test; # *p* < 0.001.

Replication kinetics of MCV19 and its I38T variant in HAE cells revealed reduced viral loads for the mutant compared to the WT at all time points, i.e., 24, 48, 72, 96, and 120 h p.i. (Figure 2A), while no significant difference was observed in viral loads for the WT and I38T variant of B/Yamagata-like virus, rg-PKT13 at the same timepoints. A similar tendency was observed for both lineages in infectious viral titers (Supplementary Material Figure S1).

### 2.5. Genetic Stability of the MCV19 I38T Variant

The MCV19 WT and I38T viruses were passaged four times in cell culture in the absence of selective pressure with BXA. Viral genes comprising the viral ribonucleoproteins (PB1, PB2, PA, and NP) were then sequenced. The I38T substitution was still present as a pure population (based on sequence chromatograms) with no other mutations. The I38T substitution is thus stable in the B/Victoria/2/1987 lineage background.

### 2.6. The MCV19 I38T Substitution Did Not Show a Significant Effect on Polymerase Activity

**Figure 3:**
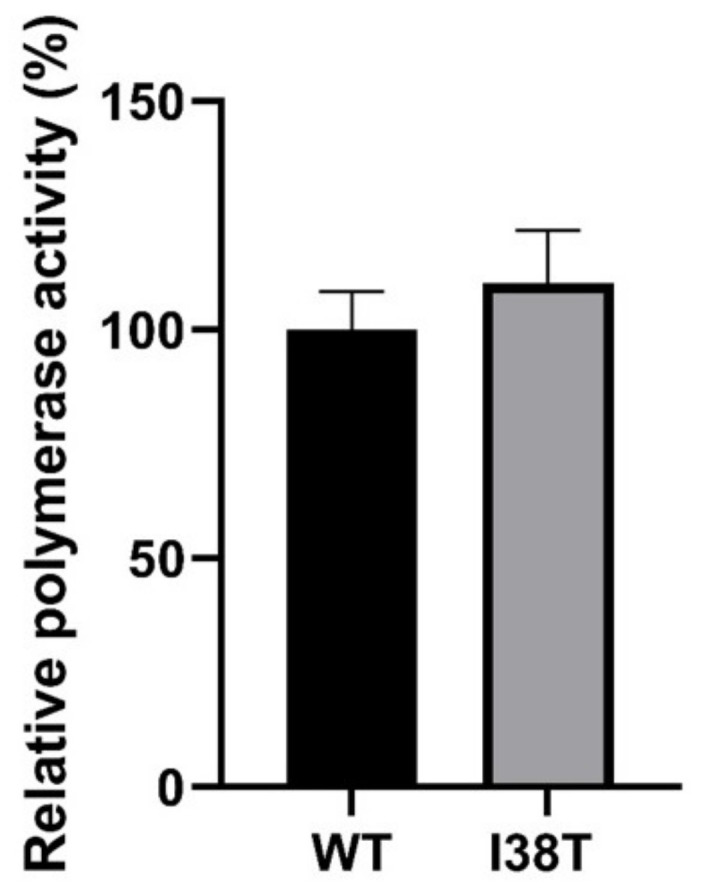
Polymerase activity of MCV19 WT and its I38T variant measured by minigenome assay. The pBZ plasmids: NP, PA, PB1, PB2, and the reporter luciferase plasmid were co-transfected in HEK293T cells. Luciferase activity was measured in supernatant after 18 h of incubation at 37 °C.

The minigenome experiment assessing the relative polymerase activity of the MCV19 WT and its PA-I38T variant showed a 10% increase for the I38T variant compared to the WT (Figure 3). However, such a difference was not statistically significant.

## 3. Discussion

The emergence of drug-resistant viruses represents a major public health problem that can compromise the clinical outcome of the newly-approved BXM. Understanding the mechanisms of resistance and the evaluation of the fitness of such variants is therefore needed to better understand their potential for dissemination among the population. In that matter, M2 channel inhibitor use is no longer recommended for the treatment of IAV infections, since all circulating IAVs are no longer susceptible to this drug due to the widespread dissemination of the S31N substitution [17].

Mutations of reduced sensitivity to BXA were first detected in phase II and III of BXM clinical trials. This resistance phenotype was explained by substitutions in the sequence of PA protein, particularly at the I38 amino acid position. Such clinical studies involved adults, adolescents, and children infected with A(H1N1) pdm09 and A(H3N2) viruses [10,11]. Very few clinical baloxavir-resistant cases involved IBV. Nonetheless, the CAPSTONE-2 phase III trial revealed that different substitutions at residue I38 (I38X) were reported in 1 of 131 enrolled subjects with an influenza B infection (lineage not determined) after a single-dose of BXM [18]. Therefore, there is a need for additional investigations of drug-resistant IBV infections and their potential for dissemination.

In this study, we passaged IBV in cell culture to induce baloxavir resistance. We and others previously demonstrated that the NA mutations induced by in vitro passages were similar to those observed in clinical cases of resistance to NA inhibitors [6,19]. Accordingly, we suggest that the in vitro selection process could help to understand the mechanism of resistance to BXA in IBV. Our in vitro selection of BXA resistance succeeded for the B/Victoria/2/1987-like isolate (MCV19) since we induced the I38T PA substitution that appeared under drug pressure and evolved to become a pure population (based on chromatogram peaks of Sanger sequencing) in presence of 200 nM of BXA. Such a mutation was not present in the viral samples recovered after the same number of passages in absence of BXA (control). By contrast, we did not succeed to induce mutations of BXA resistance in the B/Yamagata/16/1988 background (PKT13). We lost the PKT13 virus at a passage of 100 nM and could not identify new mutations in PA, PB1 and PB2 (by Sanger sequencing) at lower BXA concentrations. Ideally, we could have started again the selection process by repeating passages several times at lower concentrations before increasing again BXA concentrations but that was not done.

We cannot exclude the possibility that the I38T substitution and other important genetic changes might have occurred at a lower frequency so that more sensitive sequencing tools would have been needed. Our work confirms that, as for seasonal influenza A(H1N1) and A(H3N2) viruses, the I38T substitution is the major marker of baloxavir resistance in IBV (B/Victoria/2/1987 lineage), highlighting the importance of such a mutation. Indeed, I38 is highly conserved within the endonuclease domain of IAV and IBV and co-crystal structural analysis showed that the I38T substitution alters the van der Waals contacts between the endonuclease and BXA, resulting in reduced stability of the drug-endonuclease linkage [11]. Notably, based on our experiments and on reports by other groups, it appears that, as for oseltamivir, the influenza B strains have higher IC_50_ values than influenza A strains [20]. On the other hand, the I38T mutation induced higher resistance levels in influenza A(H1N1) pdm09 and A(H3N2) viruses (100-fold and 200-fold increases in IC_50_ values, respectively) compared to influenza B (<25-fold increase) [13,15]. Contrasting with the resistance to BXA, the NA mutations that confer resistance to NA inhibitors are rather type- and subtype-specific. For example, the D198N and R152K substitutions in the NA of IBV confer resistance to oseltamivir and zanamivir, while oseltamivir resistance is predominantly mediated by the H274Y substitution in A(H1N1) background and by the R292K and E119V substitutions in A(H3N2) subtype [21,22,23].

We previously rescued and characterized the recombinant influenza B/Phuket/3073/2013 (rg-PKT) WT virus and different PA variants (I38T/M and E23K) in vitro and in experimentally infected mice ^13^. Notably, we found that the I38T variant retained ≈ 80% of its polymerase activity and grew at similar viral titers when compared to the WT counterpart. The replication kinetics of rg-PKT13 WT and I38T on HAE also showed no impairment in viral fitness (Figure 2B). In mice, the WT and I38T recombinants induced mortality rates of 60 and 40%, respectively, and similar lung viral titers were obtained for the two groups at days 3 and 6 p.i. [13]. In the present work, we failed to induce BXA resistance in the rg-PKT13 background by in vitro passages. Additional passages and different selection approaches including other cell lines could be more successful for that purpose.

Here, we focused on investigating the impact of I38T in the IBV B/Victoria/2/1987-like background using a contemporary clinical isolate (MCV19) that was subjected to passages under in vitro BXA pressure. As we did with the B/Yamagata/16/1988 lineage, we further developed a reverse genetics system for the B/Victoria/2/1987 lineage using the current vaccine strain (B/Washington/02/2019) and confirmed the effect of the I38T PA substitution on the BXA resistance phenotype (Table 1). Then, we evaluated the fitness of MCV19-I38T in ST6GalI-MDCK cells and HAE cells. Our findings demonstrate that the replication of MCV19-I38T was impaired at 24 h p.i. in the ST6GalI-MDCK cell line whereas significantly reduced viral loads were obtained at all time points in the HAE model (Figure 1 and Figure 2A). Thus, viral fitness of I38T mutants could vary according to the B lineage.

Notably, the impact of PA mutations that confer resistance to BXA on virus transmissibility could not be reliably predicted by in vitro replicative properties. It remains interesting to assess the impact of the I38T PA substitution on the viral fitness using an appropriate animal model. A recent study demonstrated that the PA-I38T substitution did not alter IAV (A(H3N2) and A/H1N) pdm09 strains) and IBV (B/Brisbane/60/2008 and B/Phuket/3073/2013 strains) contact or airborne transmissibility in ferrets despite attenuated replicative capacities in MDCK cells [24]. Another report confirmed that the recombinant B/Phuket/3073/2013 I38T PA mutant could be transmitted between ferrets with or without BXM treatment [25]. Using a competitive mixture ferret model, Lee and collaborators confirmed that seasonal IAV I38T PA variants replicate and transmit between ferrets; however, a relative fitness cost, particularly important in the A(H1N1) pdm09 background, could be observed [26]. Thus, the impact of the I38T substitution likely varies according to the virus type, subtype, or lineage background.

In conclusion, the impact of baloxavir resistance mutations still needs to be further investigated in IBV using different animal studies to assess infectivity (mouse models) and/or transmissibility (guinea pig or ferret models). There is also a need to select and standardize the most efficient in vitro methodologies to predict in vivo viral fitness for PA mutants. It is also of importance to monitor the emergence of influenza viruses with a phenotype of reduced drug susceptibility, especially I38X variants that are considered to be the main baloxavir-resistant marker. In that regard, pyrosequencing is an interesting tool to rapidly detect the presence of this substitution in clinical specimens [27].

## 4. Materials and Methods

### 4.1. Cells, Antivirals, and Viruses

Madin–Darby canine kidney (MDCK) cells overexpressing α2,6 sialic acid receptors (ST6GalI-MDCK, kindly provided by Y. Kawaoka from the University of Wisconsin-Madison, WI) were maintained in minimum essential medium (MEM) supplemented with 10% fetal bovine serum (FBS) (Invitrogen, Carlsbad, CA, USA) and 7.5 µg/mL puromycin (Sigma, Oakville, ON, Canada). MDCK and human embryonic kidney 293T cells (ATCC) were maintained in MEM and Dulbecco’s modified Eagle’s medium (DMEM) (Invitrogen, Carlsbad, CA) supplemented with 10% FBS, respectively. MucilAir is a reconstituted human airway epithelium (HAE) from human nasal primary cells collected from a pool of 14 donors. HAE was purchased from Epithelix (Geneva, Switzerland) and cultured at the air–liquid interface with MucilAir culture medium in Costar Transwell inserts (Corning, NY, USA) according to the manufacturer’s instructions. Baloxavir acid (BXA) was synthesized at Shionogi & Co., Ltd., Osaka, Japan. Drug dilutions were made in sterile water.

Influenza B/Phuket/2073/2013 (PKT13), the current B/Yamagata/16/1988-like vaccine strain, was obtained from the National Institute for Biological Standards and Control (NIBSC, code 14/236) and used for the rescue of the recombinant PKT13 virus (rg-PKT13) by reverse genetics [28]. Influenza B/Quebec/MCV-11/2019 virus (MCV19), a B/Victoria/2/1987-like isolate was recovered from a patient at the CHU de Québec (Québec City, QC, Canada) during the 2019–2020 season. Viral stocks were prepared by infecting ST6GalI-MDCK cells in MEM supplemented with 1 µg/mL tosyl phenylalanyl chloromethyl ketone (TPCK)-treated trypsin (Sigma, Oakville, ON, Canada) before titration by plaque assays in ST6GalI-MDCK cells.

### 4.2. Development of a Reverse Genetics System for Influenza B/Victoria/2/1987-like Viruses

To confirm the role of eventual drug-induced mutations in the influenza B/Victoria/2/1987-like background, we developed a reverse genetics system using influenza B/Washington/02/2019 strain (NIBSC, code 19/190) which is the current vaccine strain. Amino acid sequences of the HA and NA proteins of MCV19 (GenBank: ON494490.1; ON494489.1) have 98.4% and 99.6% matched identities with those of B/Washington/02/2019. Reverse genetics and PCR-mediated mutagenesis were performed using the bidirectional pBZ plasmids (kindly provided by B. Zhou from the University of New York, NY, USA). Co-transfection experiments were performed as previously described [29]. Briefly, a co-culture of 2.5 × 10^5^ MDCK and 7.5 × 10^5^ 293T cells was grown in a 6-well plate with DMEM supplemented with 10% FBS and Hepes at 37 °C for 48 h. One µg of each plasmid and lipofectamine 2000 (3 µL/µg DNA) in 1.2 mL optiMEM (Gibco; ThermoFisher Scientific, Waltham, MA, USA) were used to transfect the MDCK/293T co-culture. After 6 h of incubation at 33 °C, the transfection medium was replaced with DMEM with 0.1% bovine serum albumin (BSA), TPCK (3 µg/mL) and Hepes. The co-culture was then incubated at 33 °C for 72 h before collecting the supernatant. Recombinant viruses were then amplified by infecting ST6GalI-MDCK confluent cells. All IBV segments were then sequenced using the Applied Biosystems 3730xl DNA Analyzer (Life Technologies Corporation, Carlsbad, CA, USA).

### 4.3. Selection of Baloxavir-Resistant IBV

To induce resistance to baloxavir in IBV, rg-PKT13, and MCV19 viruses were submitted to repeated passages in presence of increasing concentrations of BXA [19]. Briefly, a confluent monolayer of ST6GalI-MDCK cells was infected with each virus starting at an MOI of 0.0001, in presence of 1 nM of BXA. The drug concentration was then slowly increased (3 nM, 10 nM, 30 nM, 100 nM, 150 nM, 200 nM, 300 nM, then 400 nM) for the subsequent passages with some of them being performed at the same level to increase viral replication. Concurrently, WT viruses were passaged in the absence of BXA as controls. Every 3 passages, the supernatant of infected cell cultures was collected, then viral RNA was isolated (Qiagen viral RNA extraction kit) and reverse-transcribed before PCR amplification of the PA gene. Amplicons were purified before sequencing with an Applied Biosystems 3730xl DNA Analyzer (Life Technologies Corporation, Carlsbad, CA, USA).

### 4.4. Determination of BXA Susceptibility

The phenotype of susceptibility to BXA was determined for baseline influenza B viruses and their PA mutants using a plaque reduction assay [13]. Briefly, confluent monolayers of ST6GalI-MDCK cells were grown in 6-well plates and then infected with approximately 50 PFUs in MEM followed by an incubation of 1 h at 33 °C with 5% CO_2_. The infection medium was then removed and the cells were overlaid with MEM containing 0.8% sea plaque agarose (Lonza, Rockland, ME, USA) supplemented with 1 µg/mL TPCK-treated trypsin (Sigma, Oakville, ON, Canada) and 3-fold serial dilutions of BXA ranging from 0 to 1215 nM. After three days of incubation at 33 °C, the overlay was removed and the cells were fixated with a formalin solution followed by staining with 0.8% crystal violet. Viral plaques were then counted for determination of the drug concentration resulting in the reduction of plaque-forming units (PFU) by 50% (IC_50_ value).

### 4.5. Replication Kinetics in ST6GalI Cells

The in vitro replicative capacity of the MCV19 isolate and its drug-induced I38T variant was determined in ST6GalI-MDCK cells. Twelve-well plates of confluent cells were washed with PBS, then infected with WT influenza B virus and its I38T variant at an MOI of 0.0001. Supernatants were collected at 12-, 24-, 48-, 72-, and 96-h post-inoculation (p.i.) and titrated by tissue culture infectious dose 50% (TCID_50_) assay with the Reed and Muench endpoint method [30].

### 4.6. Replication Kinetics in HAE Cells

The apical layers of HAE were gently washed with warm opti-MEM before infection. The epithelium was infected at an MOI of 0.002 with MCV19 or rg-PKT13 and their corresponding PA I38T variants. Viruses were adsorbed at 33 °C under a 5% CO_2_ atmosphere for 30 min. Then, the inoculum was harvested and the HAE was cultured at the air–liquid interface. Apical washes were collected at 0, 12, 24, 48, 96, and 120 h p.i. RNA extraction was performed from 90 µL of HAE apical washes using the MagNA Pure LC (Total nucleic acid isolation kit, Roche Molecular System, Laval, QC, Canada). Reverse transcription-quantitative PCR (qRT-PCR) assay was performed using primers targeting the influenza NS1 gene of IBV (available upon request). This assay was performed with the QuantiTect Virus + ROX Vial Kit (Qiagen, Toronto, ON, Canada) on a LightCycler^®^ 480 system (Roche Molecular System).

### 4.7. In Vitro Genetic Stability

The genetic stability of the WT MCV19 virus and its drug-selected I38T mutant was assessed after 4 serial passages in ST6GalI-MDCK cells without BXA. A confluent monolayer of ST6GalI-MDCK in 12-well plates was infected at an MOI of 0.0001 per well. After an incubation of 1 h at 33 °C, the infection medium was removed and replaced with fresh MEM supplemented with 1 µg/mL TPCK. After 3–4 days of incubation, the supernatant was collected and viral RNA was extracted, reverse transcribed, and PCR-amplified before sequencing the PA, PB1, PB2, and NP genes with an Applied Biosystems 3730xl DNA Analyzer (Life Technologies Corporation, Carlsbad, CA, USA).

### 4.8. Minigenome Assay for Polymerase Activity

A minigenome assay was performed to assess and compare the polymerase activities of MCV19 and its I38T PA mutant. Briefly, the Gaucia luciferase reporter plasmid (pHH21-vM-Luc) was co-transfected with pBZ plasmids containing NP, PB1, PB2, and PA genes of MCV19 in 293T cells using Lipofectamine 2000 reagent (Invitrogen, Carlsbad, CA, USA). After 18 h of incubation at 37 °C, supernatants were harvested and luminescence was measured with a multilabel plate reader (Victor; PerkinElmer, Waltham, MA, USA), using an acquisition period of 1 s.

### 4.9. Statistical Analysis

Statistical analyses and graphs were conducted with version 9.1.0 of GraphPad Prism. Statistical significance is defined as *p*-value < 0.05. Student’s unpaired *t*-tests were used to compare different experimental conditions in replication kinetics experiments and enzymatic assay.

## Figures and Tables

**Table 1:** Baloxavir susceptibilities of influenza B clinical and recombinant viruses measured by plaque reduction assay.

Virus	Baloxavir IC_50_ (nM) ^a^	Fold Change ^b^
B/Quebec/MCV-11/2019	53.2 ± 7	1
B/Quebec/MCV-11/2019-I38T	731.3 ±239	13.7
rg-B/Washington/02/2019	31.6 ± 1.9	1
rg-B/Washington/02/2019-I38T	676 ± 144.2	21.3

^a^ Mean BXA IC_50_ values ± SD were determined in duplicate by plaque reduction assay performed on ST6GalI-MDCK cells. ^b^ The fold change was calculated by comparing the IC_50_ value of the mutant to the respective WT virus.

## Data Availability

Not applicable.

## Supplementary Materials

The following information can be downloaded at: https://www.mdpi.com/article/10.3390/pathogens11091048/s1. Figure S1: Infectious viral titers in vitro. Apical washes were harvested at indicated timepoints. Viral titers were determined on MDCK-ST6GalI cells. (A) MCV WT and I38T viruses; (B) rg-PKT13 WT and I38T viruses. Each experiment was performed in duplicate.
