# Supplementary material for: Generation and Characterization of Drug-Resistant Influenza B Viruses Selected In Vitro with Baloxavir Acid
Source: Pathogens. 2022 Sep 15;11(9):1048. doi: 10.3390/pathogens11091048 (PMC9505253; doi:10.3390/pathogens11091048)
Supplement: Supplementary file 1 [file pathogens-11-01048-s001.zip › pathogens-1839868-supplementary.pdf]

Supplementary data

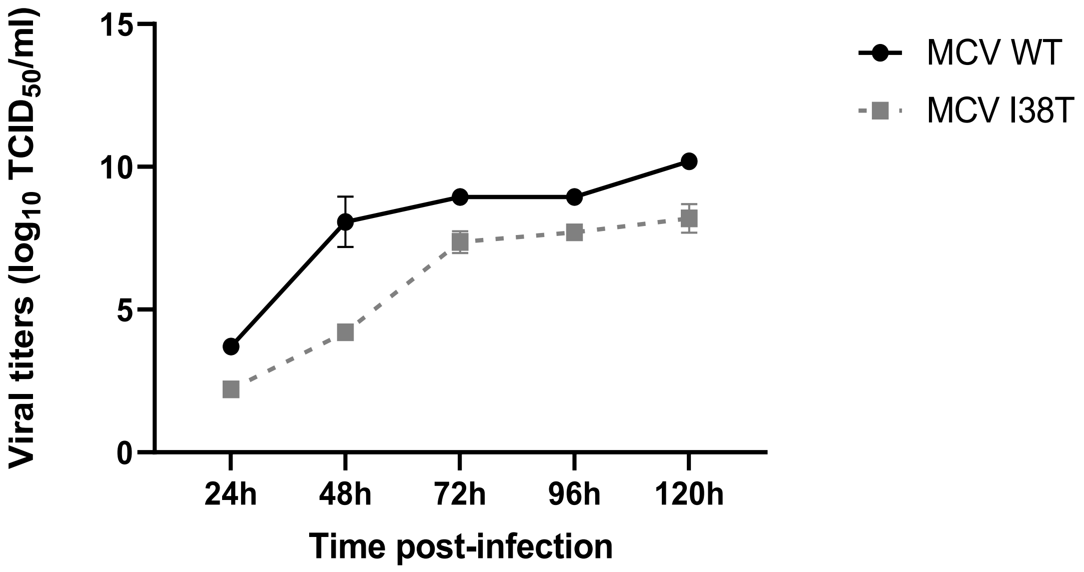

A

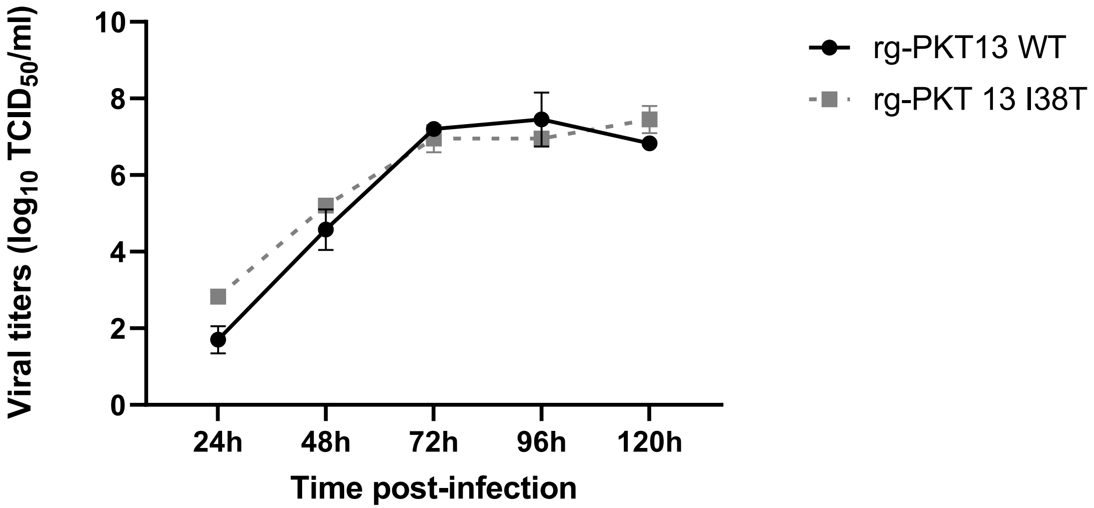

B

Supplemental Figure S1. Infectious viral titers *In vitro*. Apical washes were harvested at indicated timepoints. Viral titers were determined on MDCK-ST6Gal1 cells. A) MCV WT and I38T viruses; B) rg-PKT13 WT and I38T viruses. Each experiment was performed in duplicate.
